# Executive Attentional Dyscontrol as a Core Cognitive and Behavioral Feature of Individuals with Obesity and Cardiovascular Disease: A Cross-Sectional Investigation

**DOI:** 10.3390/brainsci13081182

**Published:** 2023-08-10

**Authors:** Giada Pietrabissa, Davide Maria Cammisuli, Federica Scarpina, Clarissa Volpi, Lia Crotti, Alessandro Mauro, Luca Alessandro Gondoni, Gianluca Castelnuovo

**Affiliations:** 1 Department of Psychology, Catholic University of Milan, 20123 Milan, Italy; giada.pietrabissa@unicatt.it (G.P.); gianluca.castelnuovo@unicatt.it (G.C.); 2 I.R.C.C.S. Istituto Auxologico Italiano, Clinical Psychology Research Laboratory, 20149 Milan, Italy; 3 I.R.C.C.S. Istituto Auxologico Italiano, Neurology and Neurorehabilitation Department, San Giuseppe Hospital, 28824 Piancavallo, VCO, Italy; f.scarpina@auxologico.it (F.S.); mauro@auxologico.it (A.M.); 4 Department of Neuroscience “Rita Levi Montalicini”, University of Turin, 10126 Turin, Italy; 5 I.R.C.C.S. Istituto Auxologico Italiano, Cardiac Rehabilitation Department, San Luca Hospital, 20149 Milan, Italy; c.volpi@auxologico.it (C.V.); l.crotti@auxologico.it (L.C.); 6 Department of Medicine and Surgery, Milano Bicocca University, 20126 Milan, Italy; 7 I.R.C.C.S. Istituto Auxologico Italiano, Cardiac Rehabilitation Department, San Giuseppe Hospital, 28824 Piancavallo, VCO, Italy; l.gondoni@auxologico.it

**Keywords:** cardiovascular disease, cardiac rehabilitation, obesity, attentional deficit, impulsiveness, anxiety, depression, quality of life

## Abstract

Executive attention as a frontal domain ability that is effective in potentially blocking distracting information, reconciling conflicts among simultaneous attentional demands, and regulating impulsive behavior may be impaired in individuals with obesity and cardiovascular disease (CVD). This study aimed (i) to explore the presence of selected cognitive (global cognitive impairment, sensitivity to interference, and attention) and psychological (quality of life, depression, anxiety, and impulsivity) dimensions and (ii) to examine the interactive relationship between attentional dyscontrol—both as a psychological and as a cognitive measure—and the above-mentioned variables in a sample of patients with CVD attending a cardiac rehabilitation program across different body mass index (BMI) levels. Clinical information of 104 patients with CVD was retrospectively collected. Participants were classified into three groups according to their BMI as follows: normal weight (NW = 30), overweight (OW = 19), and obese (OB = 55). Individuals with CVD and a higher BMI showed problems in controlling executive attention—through both neuropsychological and behavioral measures. Specifically, OB patients demonstrated reduced sensitivity to cognitive interference, lower capabilities in divided attention during visual-tracking tasks, and greater impulsivity compared to NW patients. This behavioral characteristic was also found to be correlated with higher levels of anxiety and depression and a lower quality of life. Implications for cognitive rehabilitation were discussed to offer directions for better management of patients with CVD and obesity.

## 1. Introduction

Cardiovascular disease (CVD) is a complex and chronic medical condition that currently accounts for 34.81% of total deaths in Italy [1,2], contributing significantly to increased hospitalization, increased healthcare expenditures, and decreased quality of life (QoL) [3]. Large clinical trials have demonstrated the success of pharmacologic and lifestyle interventions in improving patient health outcomes. However, patients with CVD usually suffer from multiple comorbidities, which affect their self-care ability [4,5].

Cognitive impairment (CI) is an important CVD-related condition, especially due to increasing incidence rates and the impact on daily life activities [6]. CI in CVD may manifest across a range of severity, with problems in memory, attention, learning, recall, motor speed, reaction times, and executive functions [7,8,9].

Decades ago, a provocative editorial in The Lancet [10] coined the term “cardiogenic dementia” to indicate a cutting-edge open research area about the relationship between CVD and CI that was subsequently shot down by Emerson and colleagues (1981). Many studies have shown that a link between CVD and CI is present, especially in the aging brain, and mainly reflects cerebral hypoperfusion as a result of insufficient blood supply to meet the neurometabolic demand [11,12,13]. Impaired cognitive functioning has been reported in 55.6% of patients with acute myocardial infarction (MI) [14]. Moreover, reports on the frequency of CI in persons with heart failure (HF) are varied, being between 25% and 75% [15,16]. Further, a recent meta-analysis of 119 studies has established the overall prevalence of CI and dementia in patients with HF as 41.42% and 19.79%, respectively [17]. Research has also shown that patients with HF report up to a four-fold higher risk of presenting with cognitive dysfunction, particularly in attentional and memory domains, when compared with the general population, and that the overall prevalence of CI in outpatients with HF is as high as 50% [18]. Deckers et al. (2017) conducted a meta-analysis of 10 prospective cohort studies, revealing a strong association between coronary heart disease (CHD) and an increased risk of CI or dementia [19]. A recent systematic review and meta-analysis of 43 cross-sectional and longitudinal studies further found that individuals with atrial fibrillation have a 36% increased risk of developing CI or dementia [20]. Moreover, a direct link between coronary artery disease (CAD) and impaired brain functioning has been demonstrated in more than one cross-sectional study [21,22], and a prospective multisite longitudinal investigation of 74 participants without CI found that CVD is associated with cognitive decline related to subclinical vascular brain injuries [23]. Notably, cognitive alterations in individuals with CVD lead to increased short-term progression to death and hospital readmission, functional disability, and reduced QoL [8,24,25], thus making the management of patients with CVD more complex [26]. Considering all this evidence, the assessment of cognitive functioning represents a crucial component of cardiovascular health.

Overall, it is well known how psychiatric symptoms, such as depression and anxiety, constitute established risk factors for CVD and are associated with both CI and poorer overall outcomes [27,28]. Furthermore, the association between type A personality characteristics and CVD has been widely studied and documented over the years [29,30]. This construct was developed by Friedman and Rosenman [31] and is defined as an unhealthy lifestyle that arises from the interaction of personality factors (i.e., impatience, impulsivity, a sense of time urgency, competitiveness, striving for achievement, aggressiveness, and restlessness) and social learning processes. A systematic review [32] showed that a type A behavior pattern (TABP) would lead to a higher risk to develop CVD via the mediating role of unhealthy behaviors. These results are supported by a subsequent study, which found that—among patients suffering from CVD—those with a TABP adhere less to healthy lifestyle indications than patients with a different profile [33].

Among the TABP dimensions, impulsivity plays a critical role in adherence behaviors [34,35,36]. Impulsivity is a personality facet reflecting lower inhibitory control associated with increased sensitivity to an immediate reward regardless of negative consequences [37], and research suggests that greater psychophysiological reactivity to stress increases the risk of CI and CVD.

However, while a considerable number of studies [38,39,40,41] have focused on the relationship between CVD and CI, and a TABP and CVD [42,43,44,45], only a few contributions [46] pertain to the link between a TABP and CI and, to an even lesser extent, the associations among a TABP, CVD, and CI. Moreover, to the best of our knowledge, no study has specifically explored the association between impulsivity, CVD, and CI domains. 

Still, there are complex bi-directional interactions between social, mental, and biomedical factors that may influence the development and progression of CVD, which—at the same time—is known to increase emotional problems and CI [47].

Among the traditional risk factors, a rising body mass index (BMI, weight in kg/height in m^2^) particularly increases cardiovascular risks [48,49], and evidence exists for the link between a higher BMI and more deficient cognitive capabilities [50,51,52,53]. However, findings are controversial: Some investigations have revealed obesity to be associated with a lower risk of cognitive decline [54,55], while others have concluded that being either obese or underweight places people at risk of lower cognitive function [53,56].

Furthermore, research notes that the detrimental effect of obesity on cognitive functioning varies according to underlying health conditions. Particularly, individuals with obesity and CVD exhibit poorer attentional abilities compared to lean individuals [57], as well as diminished executive domain functioning in terms of planning, organizing, impulsivity, and attention [58,59].

A higher BMI and obesity have also been related to psychiatric disorders [60], including depression, anxiety, and poorer inhibitory control [61], impaired decision making [62], and impulsive traits [63].

In contrast, people with a higher expression of impulsivity, indeed, commonly exhibit higher food intake and body weight than their counterparts, and they typically experience more difficulty in achieving goals [64].

These considerations call for new steps toward identifying potential variables to address for the prevention and treatment of patients with CVD that would consider not only traditional medical (i.e., comorbid health conditional—BMI) and neuropsychological variables but also the relationship between them.

This retrospective study aimed to address—for the first time—gaps in the literature by exploring selected cognitive (sensitivity to interference and divided attention in visual tracking) and psychological (QoL, depression, anxiety, and impulsivity) dimensions in a sample of patients with CVD across different BMI levels. It also intended to examine the interactive relationship between attentional dyscontrol—both as a psychological and as a cognitive measure—and the above-mentioned variables in patients with CVD across BMI levels.

It was hypothesized that individuals with CVD and obesity would display greater attentional defects (in terms of both cognitive efficiency and behavioral control), lower QoL, and higher anxiety and depressive symptoms than those with overweight. It was also hypothesized that patients with CVD also presenting as overweight would exhibit higher attentional dyscontrol and worse psychological parameters than their normal-weight counterparts.

## 2. Materials and Methods

### 2.1. Participants

This was a retrospective study covering the years from 2017 to 2019. 

Consecutive patients with CVD referred to the IRCCS Istituto Auxologico Italiano, San Giuseppe Hospital in Oggebbio (Verbania, Italy), and the San Luca Hospital (Milan, Italy) to attend a cardiac rehabilitation (CR) program were screened for admission into the study. Inclusion criteria were age ≥ 18 years; Italian nationality; CVD in the medical history—including previous myocardial infarction, coronary angioplasty, or coronary artery bypass; heart failure with either reduced or preserved ejection fraction; and valvular heart disease. Every patient was in a clinically stable condition, and those who had recent (less than one month) acute events were excluded.

Another reason for the exclusion of participants from the study was the presence of visual impairments that would affect neuropsychological and psychological testing.

Informed consent was obtained from all subjects involved in the study. The study was conducted following the Declaration of Helsinki and approved by the Institutional Review Board (or Ethics Committee) of the IRCCS Istituto Auxologico Italiano (ID: 03C202_2002).

### 2.2. Measures

Socio-demographic and biomedical information, including marital status, education, employment, smoking status, and body mass index (BMI), at inclusion to CR was retrieved from the patients’ medical records. Moreover, the Italian translation and cultural adaptation of the following self-report measures of psychological constructs and neuropsychological tests were individually administered, respectively, by a certified psychologist and a neuropsychologist within the first week of the CR period. Remarkably, executive attention was particularly assessed using the Stroop Test and the Trail Making Test as neuropsychological measures and the Barratt Impulsiveness Scale as a psychological measure in order to detect both cognitive and behavioral features. 

#### 2.2.1. Psychological Measures

The *Hospital Anxiety and Depression Scale (HADS)* was used to measure the levels of anxiety (HADS-A) and depression (HADS-D) experienced by the patients [65,66] through a 14-item scale ranging from 0 to 3. The HADS questionnaire has been validated in many languages, countries, and settings, including general practice and community settings, showing good validity and reliability across studies [67]. The HADS measures symptoms of anxiety (7 items) and depression (7 items). Items are rated using a 4-point (0–3) scale, with greater scores indicating elevated distress. Scores for each subscale range from 0 to 21 and can be categorized as normal (0–7), mild (8–10), moderate (11–14), or severe (15–21).

The *Quality of Life Index-Cardiac Version (QLI)* was used to evaluate the perceived QoL of the study participants across four specific domains: (i) health and functioning, (ii) social and economic aspects, (iii) psychological and spiritual status, and (iv) family and relationships [68]. The scale comprises 36 items used to measure individuals’ levels of satisfaction (part 1) and importance (part 2) regarding various aspects of life on a 6-point Likert scale ranging from 1 = very dissatisfied/unimportant to 6 = very satisfied/important. Scores from parts 1 and 2 are combined, and higher scores represent higher satisfaction and importance. Scores for each subscale are transformed to a scale of 0–30. The QLI is a well-established instrument with substantial evidence of reliability, validity, and sensitivity. 

The *Barratt Impulsiveness Scale (BIS-11)* [69,70] is one of the most widely cited instruments for the assessment of the personality/behavioral construct of impulsiveness. It is a 30-item self-report questionnaire describing common impulsive behavior and preference. Impulsiveness is a multi-faceted construct, and its total score and three second-order factors (attentional impulsiveness, AI—or a lack of cognitive persistence with an inability to tolerate complexity; motor impulsiveness, MI—or acting on the spur of the moment; and no-planning impulsiveness, NpI—or a lack of a sense of the future) are assessed on a 4-point scale, with higher scores indicating greater impulsiveness. Patton et al. reported internal consistency coefficients for the BIS-11 total score that range from 0.79 to 0.83 for separate populations of undergraduates, substance-abuse patients, general psychiatric patients, and prison inmates [69].

#### 2.2.2. Neuropsychological Measures

The *Mini-Mental State Examination (MMSE)* [71,72,73] was used to check for global cognitive impairment (problems with thinking, communication, understanding, and memory) through a set of 11 questions and a series of tasks, such as memorizing a few objects and then repeating the list back later, copying a drawing, writing a short sentence that is grammatically correct, and correctly identifying the current day of the week, followed by the date, the month, the season, and the year. It takes about 5 to 10 min to be completed. The maximum score for the MMSE is 30, and a score below 24 usually indicates possible cognitive impairment.

The *Stroop Test* [74,75,76] was adopted to measure sensitivity to interference. This test evaluates the ability of switching from one perceptual set to another one in relation to the variation of the stimulation by suppressing habitual responses (i.e., reading words presented on a sheet) rather than naming the color in which they have been printed (e.g., the word “RED” written in blue color), the so-called “Stroop effect”. The examinee is provided with three stimulation sheets: The first one consists of a list of color names, the second one consists of a list of colored dots, and the third one represents the “Stroop effect” (i.e., a list of color names printed in different inks). The examinee is required to read aloud as soon as possible the stimuli presented on the three sheets. The examiner is equipped with a stopwatch and a grid to register the examinee’s mistakes. The interference time (calculated with the following formula: [III − (I + II)/2], where III, II, and I are equal to seconds for completing each task) and the interference error (calculated with the following formula: [III − (I + II)/2], where III, II, and I are the number of mistakes in each task) are computed. High scores indicate poor performance. 

The *Trail Making Test (TMT)* [77]—as a measure of divided attention (task switching)—provides information about individuals’ search speed in a visual-tracking tasking and task-switching ability [78,79]. It comprises two parts: the TMT-A and the TMT-B—both asking a person to draw a line between 24 consecutive circles that are randomly arranged on a page. The TMT-A uses all numbers, whereas the TMT-B alternates numbers and letters, requiring the participants to switch between numbers and letters in consecutive order. The time required to complete the test—including correction of errors prompted by the examiner—is registered in seconds; participants have a maximum time of 5 min to complete the task. High scores indicate poor performance.

### 2.3. Statistical Analysis

The age of the three different groups was compared using a one-way ANOVA, while for categorical variables (i.e., gender, education, marital status, employment, smoking), Pearson’s chi-square tests were used. The normality of the distribution of the continuous variables was assessed using the Shapiro–Wilk test, and only scores on the BIS-11 were normally distributed. A one-way ANOVA with Bonferroni post hoc comparison was then run to test differences among groups on the BIS-11, whilst non-parametric tests (i.e., the Kruskal–Wallis test first and the Mann–Whitney U test then with Bonferroni correction) were used to compare groups’ performances on the remaining psychological and neuropsychological scores. Statistical significance was considered when *p*-value < 0.05. A Spearman rank correlation was also performed to further investigate the association between the above-mentioned measures. All analyses were run in Statistical Package for the Social Sciences (SPSS) software (version 20.0; SPSS, Inc., Chicago, IL, USA) for Windows. 

## 3. Results

### 3.1. Demographic and Clinical Information 

A total of 104 consecutive patients were recruited. The patients were classified into three groups—normal weight (NW) (n = 30), overweight (OW) (n = 19), and obese (OB) patients (n = 55)—according to the body mass index (BMI) (WHO, 1995) values of 18.5–24.9, 25.0–29.9, and ≥30.0, respectively.

Descriptive statistics are shown in Table 1, Table 2 and Table 3. The three groups were matched in terms of age (F(_2,101_) = 0.882; NW, 62.87 ± 12.88; OW, 62.68 ± 10.76; OB, 60.11 ± 8.38), global cognition (MMSE scores: χ^2^ = 0.990, *df* = 2, *p* = 0.609; NW mean rank = 56.63; OW mean rank = 53.21; OB mean rank = 50.00), marital status (χ^2^ = 5.795, *df* = 6, *p* = 0.447), and smoking (χ^2^ = 9.087, *df* = 4, *p* = 0.059) but not for gender (χ^2^ = 13.955, *df* = 2, *p* = 0.001), education (χ^2^ = 26.562, *df* = 8, *p* = 0.001), and employment (χ^2^ = 14.749, *df* = 8, *p* = 0.022).

### 3.2. Differences among Groups in Psychological Measures and Neuropsychological Measures

The three groups reported significant performances on the BIS-11 total score (F(_2,101_) = 4.074) and the BIS-AI score (F(_2,101_) = 8.590). No significant differences were found in the other BIS-11 subscales (i.e., MI and NpI) (*p* = n.s.). Post hoc comparisons showed that OB patients performed significantly worse than the NW group on the BIS-11 total score (*p* = 0.045) and the BIS-AI subscale (*p* < 0.001). No other significant difference was found when performances of NW vs. OW and OW vs. OB patients were compared on these measures (*p* = n.s.).

Regarding the HADS, no differences were found when comparing the scores obtained by the groups (*p* = n.s.). Conversely, the Kruskal–Wallis test detected significant differences between groups in the QLI-total score (χ^2^ = 10.537, *df* = 2, *p* = 0.003; OB mean rank = 43.25; OW mean rank = 66.97; NW mean rank = 60.28) and the health and functioning (χ^2^ = 10.378, *df* = 2, *p* = 0.006; OB mean rank = 43.74; OW mean rank = 66.26; NW mean rank = 59.85), social and economic aspects (χ^2^ = 13,836, *df* = 2, *p* = 0.001; OB mean rank = 42.48; OW mean rank = 66.26; NW mean rank = 59.85), and family and relationship (χ^2^ = 9.080, *df* = 2, *p* = 0.001; OB mean rank = 43.34; OW mean rank = 57.71; NW mean rank = 62.62) subscales. Therefore, the Mann–Whitney U test was run, and significant performance differences were found: OB patients obtained a significantly lower score than NW patients in the family and relationship QLI dimension (*p* = 0.004). They also had lower values than OW patients in the QLI-total score (*p* = 0.004), the health and functioning dimension (*p* = 0.007), and the social and economic aspects dimension (*p* = 0.001).

Regarding cognitive functioning, the Kruskal–Wallis test detected significant differences among the groups in the Stroop interference/time (χ^2^ = 61.306, *df* = 2, *p* < 0.001; OB mean rank = 70.96; OW mean rank = 32.53; NW mean rank = 27.10), the TMT-A (χ^2^ = 56,738, *df* = 2, *p* < 0.001; OB mean rank = 72.30; OW mean rank = 34.29; NW mean rank = 26.68), and the TMT-B (χ^2^ = 43,644, *df* = 2, *p* < 0.001; OB mean rank = 69.96; OW mean rank = 35.33; NW mean rank = 27.57). Therefore, the Mann–Whitney U test was run, and significant performance differences were found: OB patients significantly showed lower performances than their NW and OW counterparts on the Stroop interference/time, the TMT-A, and the TMT-B (*p* < 0.001), but no significant difference was found when NW and OW patients were compared on these measures (*p* = n.s).

### 3.3. Correlations between Psychological Dimension and Neuropsychological Measures across the Different Levels of Body Mass Index

When we considered OB patients, the score relative to the BIS-11 was positively associated with the HADS-A (rho = 0.359, *p* < 0.01), HADS-D (rho = 0.466, *p* < 0.01), and HADS-total (rho = 0.442, *p* < 0.01) scores and negatively associated with the QLI-total score (rho = −0.525, *p* < 0.01) and the health and functioning (rho = −0.474, *p* < 0.01), social and economic aspects (rho = −0.400, *p* < 0.01), psychological and spiritual status (rho = −0.317, *p* < 0.05), and family and relationship (rho = −0.304, *p* < 0.05) subscales. Accordingly, the score obtained by OB patients on the BIS-AI was positively associated with the HADS-A (rho = 0.606, *p* < 0.01), HADS-D (rho = 0.490, *p* < 0.01), and HADS-total (rho = 0.603, *p* < 0.01) scores and negatively associated with the QLI-total score (rho = −0.525, *p* < 0.01) and the health and functioning (rho = −0.474, *p* < 0.01), social and economic aspects (rho = −0.483, *p* < 0.01), psychological and spiritual status (rho = −0.423, *p* < 0.01), and family and relationship (rho = −0.317, *p* < 0.01) subscales.

The score obtained by OW patients on the BIS-AI was positively correlated with the HADS-A (rho = 0.584, *p* < 0.01) and HADS-total (rho = 0.558, *p* < 0.05) scores and negatively correlated with the QLI-total score (rho = −0.520, *p* < 0.05) and the health and functioning (rho = −0.491, *p* < 0.05) and psychological and spiritual status (rho = −0.466, *p* < 0.5) subscales.

Lastly, the score obtained by NW patients on the BIS-AI was negatively correlated with the QLI-total score (rho = −0.520, *p* < 0.01) and the health and functioning (rho = −0.491, *p* < 0.01) subscale. Moreover, the BIS-AI was positively correlated with the Stroop interference/time (rho = 0.412, *p* < 0.01), the TMT-A (rho = 0.424, *p* < 0.01), and the TMT-B (rho = 0.357, *p* < 0.001) in the sample as a whole.

## 4. Discussion

Attentional/executive abilities are of particular interest in understanding brain–behavior relationships in obesity and CVD, as they have a substantial impact on the everyday life of the sufferers. Frontal skills include different high-order cognitive abilities, such as manipulating information and directing the focus of attention necessary for a wide inventory of daily life activities, including the capability to guide people toward healthy choices. Further, greater impulse control may lead individuals with obesity and CVD toward more adaptive behaviors.

Moreover, given the documented relationship between a higher weight and cognitive and behavioral difficulties in individuals with obesity, this study for the first time aimed to explore the differences in executive/attentional skills in a sample of inpatients undergoing CR across BMI levels.

According to our hypothesis, results showed that a higher BMI in patients with CVD entails difficulties in executive attentional control, both as a neuropsychological (i.e., TMT and Stroop Test) and a behavioral (BIS-11 questionnaire) feature. Specifically, in the sample, OB patients showed a lower level of sensitivity to cognitive interference, as well as lower abilities in divided attention during visual-tracking tasks than NW patients and greater impulsivity than their counterparts. This behavioral feature also correlated with higher anxiety and depression levels and lower QoL. The findings of this study are in line with those of previous research that showed associations between weight gain and subjective experiences of anxiety, depression, and decreased QoL [60,61] and underline that the above-mentioned psychological dimensions also play a pivotal role in obesity management.

Also, this study corroborated the conclusions reached by previous research that individuals with obesity [50,51,52,53] and CVD [57,58,59] exhibit poorer attentional/executive abilities. These cognitive defects involve difficulties in identifying and planning appropriate choices for situations that extend the risk of eating inappropriately and reduce the possibility of undertaking healthy lifestyles, as well as difficulties in emotional control of stimulations from the environment. In addition to the decision-making deficit, individuals with obesity often present with problem-solving difficulties [80]. Cognitive rehabilitation programs in the case of obesity have demonstrated positive changes in cognitive flexibility and divided attention ability after training sessions [81]. Therefore, in the context of CR programs, specific interventions, such as Goal Management Training (GMT) [82], could be integrated to increase executive attentional control through compensatory learning strategies, primarily addressing problem-solving and inhibition skills. Taken together, the ultimate goal of these actions is the promotion of self-management abilities that are of fundamental importance in persons suffering from chronic conditions.

Despite the innovative preliminary results on the cognitive and behavioral aspects of obesity patients with CVD undergoing a CR program, this study presents some limitations. First, the recruited sample consisted of patients with different CVD conditions, but it was not possible to retrospectively retrieve the specific patients’ diagnoses—and this did not allow for the investigation of diagnosis-related specificities of the sample. Second, BMI groups were unbalanced. Also, a more comprehensive neuropsychological evaluation, including other cognitive domains frequently involved in CVD and obesity, such as planning and decision making, should be considered in future investigations. Lastly, the study design did not allow authors to follow the progression of executive/attentional deficit, and longitudinal investigations should be performed, given that the literature reports controversial findings for the trajectories of cognitive decline in obesity in [53,54,55,56]. Future studies should address the above-mentioned limitations, also including participants with obesity but not suffering from a cardiovascular problem.

## 5. Conclusions

Executive attentional dyscontrol represents a core cognitive and behavioral feature of patients with CVD also presenting with obesity. These domains should, therefore, be considered and assessed within the context of CR to promote targeted interventions aimed to increase cognitive function and improve lifestyle self-management in the ecological context where persons suffering from chronic conditions are often unable to make appropriate choices based on contextual information and sustain healthy habits in the long term.

## Figures and Tables

**Table 1:** Socio-demographic characteristics of the three groups.

Group	Age (Mean ± SD)	Gender (n)	Education (n)	Marital Status (n)	Employment (n)	Smoking (n)
**NW** (n = 30)	62.87 ± 12.88	M:F = 12:18	Primary school = 3 Middle school = 7 Secondary school = 18 University = 1 Post-graduate level = 1	Single = 7 Married = 15 Separated = 3 Widowed = 5	Student = 0 Worker = 1 Housewife = 14 Unemployed = 2 Retired = 14	In the past = 14 Yes = 2 No = 14
**OW** (n = 19)	62.68 ± 10.76	M:F = 13:6	Primary school = 1 Middle school = 2 Secondary school = 10 University = 6 Post-graduate level = 0	Single = 2 Married = 12 Separated = 3 Widowed = 2	Student = 0 Worker = 1 Housewife = 8 Unemployed = 0 Retired = 10	In the past = 6 Yes = 2 No = 11
**OB** (n = 55)	60.11 ± 8.38	M:F = 44:11	Primary school = 14 Middle school = 32 Secondary school = 48 University = 9 Post-graduate level = 1	Single = 5 Married = 34 Separated = 11 Widowed = 5	Student = 0 Worker = 16 Housewife = 12 Unemployed = 4 Retired = 23	In the past = 9 Yes = 6 No = 40

Notes: NW = normal weight; OW = overweight; OB = obese.

**Table 2:** Scores obtained on the psychological measures by the three groups, with reported statistical significance.

Psychological Measures (Mean ± SD)	NW (n = 30)	OW (n = 19)	OB (n = 55)
HADS-total	Mean ± SD 8.03 ± 7.22 Median 2.00	Mean ± SD 7.00 ± 6.29 Median 5.00	Mean ± SD 10.56 ± 7.06 Median 10.00
HADS-A	Mean ± SD 4.63 ± 4.42 Median 3.50	Mean ± SD 4.05 ± 4.02 Median 2.00	Mean ± SD 5.97 ± 4.16 Median 5.06
HADS-D	Mean ± SD 3.40 ± 3.27 Median 2.00	Mean ± SD 2.97 ± 2.89 Median 2.00	Mean ± SD 4.63 ± 3.61 Median 4.00
QLI-total	Mean ± SD 23.47 ± 2.95 Median 23.63	Mean ± SD 24.03 ± 2.75 Median 24.51	Mean ± SD 20.88 ± 4.17 Median 21.47
QLI-Hlth/Func	Mean ± SD 22.68 ± 3.53 Median 22.12	Mean ± SD 23.31 ± 2.82 Median 23.96	Mean ± SD 19.99 ± 4.55 Median 20.40
QLI-Socioec	Mean ± SD 23.59 ± 3.72 Median 24.50	Mean ± SD 24.70 ± 2.84 Median 24.16	Mean ± SD 21.19 ± 4.02 Median 21.78
QLI-Psych/spir	Mean ± SD 23.11 ± 3.63 Median 22.67	Mean ± SD 23.56 ± 4.09 Median 23.78	Mean ± SD 20.81 ± 5.38 Median 21.64
QLI-Family	Mean ± SD 26.23 ± 4.35 Median 27.60	Mean ± SD 25.76 ± 4.13 Median 27.60	Mean ± SD 23.62 ± 4.87 Median 24.98
BIS-Total	Mean ± SD 57.53 ± 8.40 Median 55.50	Mean ± SD 57.52 ± 7.62 Median 57.00	Mean ± SD 62.11 ± 8.14 Median 61.00
BIS-AI	Mean ± SD 13.23 ± 2.43 Median 13.00	Mean ± SD 14.84 ± 3.38 Median 15.00	Mean ± SD 16.09 ± 3.20 Median 16.00
BIS-MI	Mean ± SD 18.76 ± 4.63 Median 18.50	Mean ± SD 17.84 ± 2.91 Median 18.00	Mean ± SD 19.94 ± 3.39 Median 20.00
BIS-NpI	Mean ± SD 25.53 ± 5.51 Median 26.50	Mean ± SD 28.84 ± 5.03 Median 26.00	Mean ± SD 26.07 ± 4.91 Median 26.00

Notes: NW = normal weight; OW = overweight; OB = obese; HADS = Hospital Anxiety and Depression Scale; A = anxiety; D = depression; QLI = Quality of Life Index-Cardiac Version. Hlth/Func = health and functioning; Socioec = social and economic aspects; Psych/spir = psychological and spiritual status; Family = family and relationship; BIS = Barratt Impulsiveness Scale; AI = attentional impulsiveness; MI = motor impulsiveness; NpI = no-planning impulsiveness; OB < NW, *p* < 0.05; OB < OW, *p* < 0.05.

**Table 3:** Scores obtained on the neuropsychological measures by the three groups, with reported statistical significance.

Groups	MMSE	Stroop Interference Time *	Stroop Interference Error	TMT-A *	TMT-B *
**NW** (n = 30)	Mean ± SD 27.46 ± 2.86 Median 29	Mean ± SD 3.93 ± 0.36 Median 4	Mean ± SD 3.03 ± 1.32 Median 4	Mean ± SD 3.33 ± 1.24 Median 4	Mean ± SD 3.41 ± 1.08 Median 4
**OW** (n = 19)	Mean ± SD 27.89 ± 1.44 Median 28	Mean ± SD 5.73 ± 5.28 Median 4	Mean ± SD 3.07 ± 1.41 Median 4	Mean ± SD 7.52 ± 11.48 Median 4	Mean ± SD 14.36 ± 34.71 Median 4
**OB** (n = 55)	Mean ± SD 27.40 ± 2.38 Median 28	Mean ± SD 24.01 ± 14.93 Median 22	Mean ± SD 3.53 ± 5.17 Median 2	Mean ± SD 40.81 ± 20.97 Median 42	Mean ± SD 76.63 ± 45.04 Median 77

Notes: NW = normal weight; OW = overweight; OB = obese; MMSE = Mini-Mental State Examination; TMT = Trail Making Test; * OB > NW and OW, *p* < 0.05.

## Data Availability

Data are available on request due to privacy and ethical restrictions.
